# The prevalence and risk factors of acute kidney injury in patients undergoing hip fracture surgery: a meta-analysis

**DOI:** 10.1080/21655979.2021.1926200

**Published:** 2021-05-26

**Authors:** Zi-Cai Li, Yan-Chuan Pu, Jin Wang, Hu-Lin Wang, Yan-Li Zhang

**Affiliations:** Department of Orthopaedics, Wuwei People’s Hospital, Wuwei, China

**Keywords:** Acute kidney injury, prevalence, risk factor, hip fracture, meta-analysis

## Abstract

Acute kidney injury (AKI) was a frequent complication following hip fracture surgery, but recent studies reported inconsistent findings. Our study was aimed at clarifying the prevalence and risk factors of AKI after hip fracture surgery. Pubmed, Embase, and Web of Science were systematically searched from the inception to March 2020 to identify observational studies investigating the prevalence and risk factors of AKI in patients undergoing hip fracture surgery. Pooled prevalence and odds ratios (ORs) with 95% confidence intervals (CIs) were estimated using a random-effects model. Publication bias was evaluated with a funnel plot and statistical test. All the statistical analyses were performed using Stata version 12.0. A total of 11 studies with 16,421 patients was included in the current meta-analysis. The pooled prevalence of AKI in patients undergoing hip fracture surgery was 17% (95%CI, 14%-21%) with substantial heterogeneity (I^2^ = 95%). Postoperative serum albumin (OR 1.80; 95%CI, 1.38–2.36) was a significant predictor for AKI. Age (OR 1.01; 95%CI, 0.95–1.07) and ACE inhibitors (OR 1.38; 95%CI, 0.92–2.07) were associated with increased the risk of AKI, but the results were not statistically significant. No significant publication bias was identified through statistical tests (Egger’s test, *p* = 0.258 and Begg’s test, *p* = 0.087). In conclusion, our findings indicated that **t**he pooled AKI following hip fracture surgery was approximately 17%. Postoperative serum albumin was a potential significant risk factor for AKI.

## GRAPHICAL ABSTRACT

**Figure:**
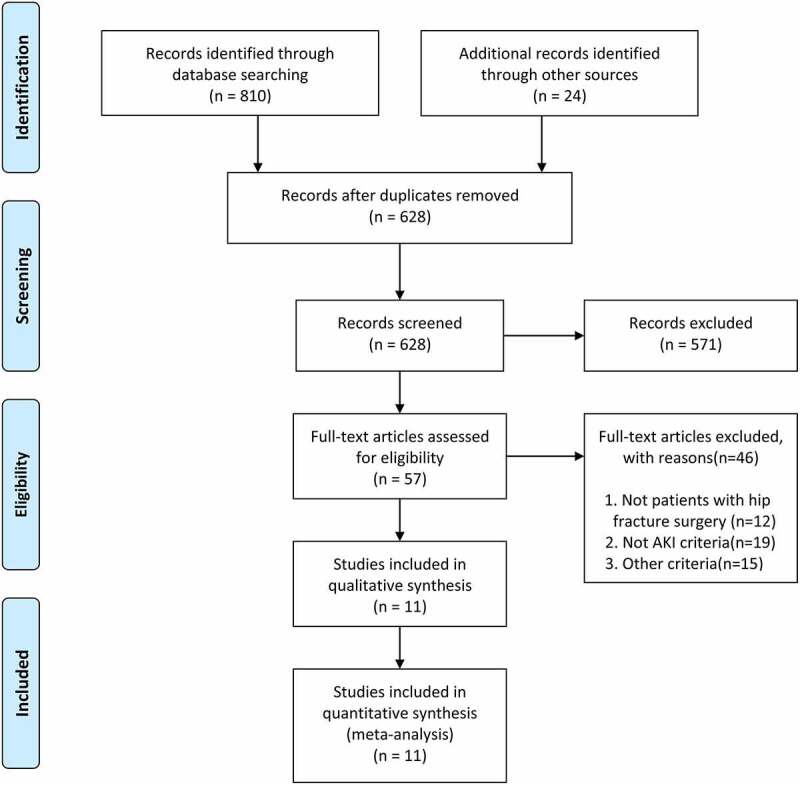

## Introduction

With the aging of the worldwide population, the prevalence of osteoporotic hip fractures increase year after year[1].^,^ [2] Accordingly, a recent statistical estimation predicts that patients undergoing hip fracture surgery may double by 2050[3]. Acute kidney injury (AKI), is a clinical syndrome featured with a sudden impairment in glomerular filtration. Every year, about 13.3 million cases are diagnosed with this intractable syndrome all over the world and it has been estimated that AKI results in considerable mortality (nearly 1.7 million deaths per year) globally [4,5]. Notably, AKI is also common in patients undergoing hip fracture surgery, which was usually associated with prolonged hospital stay, increased morbidity and mortality, and impaired quality of life [6,7]. Therefore, to adequately understand the landscape of prevalence and risk factors of AKI may be conducive to improve prognosis of patients undergoing hip fracture surgery.

Actually, the prevalence of AKI after hip fracture surgery was inconsistent across several published studies, ranging from 5% to 60%. This controversiality may originate from the heterogeneous populations, different AKI definition, and inconsistent follow-up durations. For clinicians, an inaccurate estimation of AKI prevalence will help them comprehensively understand an overview of the disease burden and evaluate whether specific treatment strategies are effective for preventing AKI. Additionally, increasing studies also revealed that some risk factors were significant predictors for AKI after hip fracture surgery, including chronic kidney disease, postoperative serum albumin, and intraoperative hypotension [6,8]. Nevertheless, there were no available studies to systematically summarize these risk factors and quantitatively assess the correlative dimension between these identified factors and AKI. Understandably, it is very necessary to illuminate the prevalence and risk factors of AKI in patients undergoing hip fracture surgery, which may provide potential individualized guidance for the prevention of AKI after hip fracture surgery.

Considering the aforementioned controversy and uncertainty, we performed a meta-analysis and systematic review to systematically investigate the prevalence and risk factors for AKI in patients undergoing hip fracture surgery, which contribute to providing the available optimum epidemiological evidence on this topic.

## Materials and methods

This meta-analysis was undertaken according to the guideline of the Meta-analysis of Observational Studies in Epidemiology (MOOSE) checklist and the Preferred Reporting Items for Systematic Reviews and Meta-Analysis (PRISMA) statement [9,10].

### Search strategy

Pubmed, Embase, and Web of Science were systematically searched from inception to March 2020 to identify observational studies that reported the prevalence and risk factors of AKI in patients undergoing hip fracture surgery. The search strategy was established using the terms of ‘acute kidney injury’, ‘hip fracture surgery’ and their variants. Also, the references of included studies and some important reviews were manually checked for any potential inclusion.

### Study selection

Studies involving the following inclusion criteria were included in the current meta-analysis:(1) observational studies, including cohort studies, case-control studies, or cross-sectional studies; (2) studies investigating the prevalence and risk factors of AKI in patients undergoing hip fracture surgery. Only studies published in English were considered. The Retrieved studies were individually evaluated for eligibility by the two investigators with discrepancies resolved through discussion.

### Data extraction and quality assessment

We applied the pre-designed table to extract the following information: first author, publication year, study period, country, operation type, case number, the number of patients with AKI, AKI definition and risk factors of AKI. The primary outcome was the prevalence of AKI after hip fracture surgery. The secondary outcomes were risk factors of AKI after hip fracture surgery. Furthermore, only ORs with 95%CIs on multivariate analysis in the included studies were extracted. The quality of the included studies was evaluated using Newcastle–Ottawa Scale (NOS) score[11]. The score system includes three dimensions involving selection criteria of participants, comparability, exposure, and outcome. Two authors independently performed data extraction and quality assessment, with inconsistency resolved by a third reviewer.

### Statistical analysis

The prevalences of AKI after hip fracture surgery were extracted from included studies. Pooled prevalences with 95% confidence intervals (CIs) were calculated using the generic inverse-variance method. The correlative dimension of risk factors with AKI was estimated as odds ratios (ORs) with 95%CIs using a random effect model when considering substantial statistical heterogeneity across the included studies. Only candidate risk factors reported in two or more eligible studies on multivariable models were considered for meta-analysis. I^2^ statistic was applied to evaluate the statistical heterogeneity across eligible studies and I^2^ > 50% was regarded as substantial heterogeneity [12,13]. Meta-regression analysis for publication time, sample size and NOS score were used to explore the potential source of heterogeneity. Sensitivity analysis was undertaken to investigate the influence of single study on the overall pooled effect by deleting one study at each step. Subgroup analyses based on region, sample size, study design, AKI Definition, and NOS score for the primary outcome were conducted to explore the prevalence of AKI in sub-populations. Publication bias was evaluated by Begg’s and Egger’s tests. P < 0.05 and asymmetric funnel plot indicated that there existed significant publication bias [14,15]. A two-sided P < 0.05 was identified as statistical significance. All the statistical analyses were performed using Stata 12.0 (Stata Corporation, College Station, TX, USA).

## Results

### Brief introduction

The current meta-analysis was aimed at exploring the prevalence and risk factors of AKI after hip fracture surgery. Pubmed, Embase, and Web of Science were systematically searched to identify observational studies investigating the prevalence and risk factors of AKI in patients undergoing hip fracture surgery. We found that a total of 11 studies with 16,421 patients were included in the current meta-analysis. The pooled prevalence of AKI in patients undergoing hip fracture surgery was approximately 17%. Postoperative serum albumin was a significant predictor for AKI. Age and ACE inhibitors were also associated with increased the risk of AKI, but the results were n’t statistically significant.

### Study selection and characteristics

**Figure 1.**
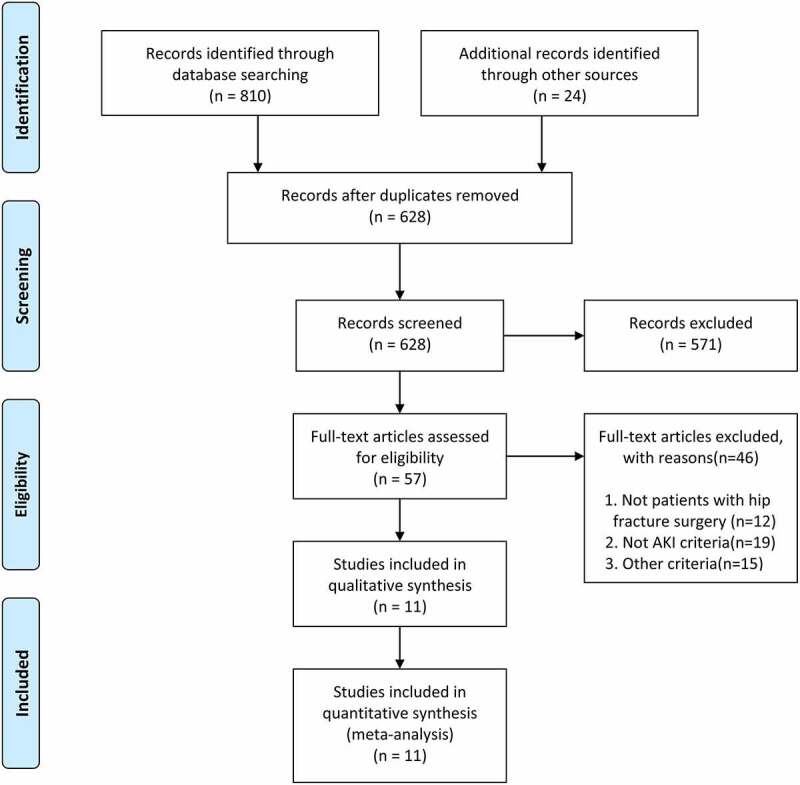
Flow diagram of the selection of studies for this meta-analysis

A total of 810 items were identified through systematically searching three databases. Also, another 24 items were obtained from other sources. After removing duplicated and irrelative items, the full texts of 57 remaining articles were screened for possible eligibility. Eventually, a total of 11 studies with 16,421 patients was included in the current meta-analysis [3,6,8,16–23]. The flow chart of study selection was summarized in Figure 1. The publication time of included studies ranged from 2010 to 2020. Seven studies were performed in Asia [6,8,17,18,20,21,23], while the other four in Europe [3,16,19,22]. Nine studies are cohort studies, another two are case-control study and retrospective descriptive study. The operation types and AKI definition of included studies were also different from each other (Table 1). The whole NOS score of included studies ranged from 6 to 8 points, which suggested that the quality of included studies was moderate to high level (Table 2).

**Table 1.** Baseline characteristics of included studies in the meta-analysis

Study/year	Study period	Country	Operation	Age(years)	N with AKI	N total	AKI Definition	Study design
Craig 2012[16]	September and November 2010	United Kingdom	Surgery for fractured neck of femur	Study group (80.3 years); control group (83.6 years)	13	100	An increase in serum creatinine by over 50% of baseline	Historical cohort study
Ulucay 2012[17]	2007–2010	Turkey	Surgery for femoral neck fracture	>65 years	25	163	AKIN classification	Prospective cohort study
Marty 2016[3]	May-October 2012	France	Hip fracture surgery	83(75–92) years	29	48	AKIN classification	Prospective cohort study
Pedersen 2016[19]	2005–2011	Denmark	Hip fracture surgery	>65 years	1717	13,529	KDIGO classification	Regional cohort study
Hong 2017[18]	2010–2012	Korea	Hip fracture surgery	>65 years	95	450	AKIN classification	Retrospective cohort study
Shin 2018[6]	2011–2016	Korea	Surgery for intertrochanteric fracture of the proximal femur	>60 years	57	481	KDIGO classification	Retrospective cohort study
Frenkelrutenberg 2019[20]	2012–2016	Israel	Surgery for fragility hip fractureS	>65 years	55	217	AKIN classification	Retrospective cohort study
Jang 2019[21]	2011–2015	Korea	Femoral neck fracture surgery	77.6(65–97) years	44	248	KDIGO classification	Retrospective cohort study
Rantalaiho 2019[22]	2017–2018	Finland	Hip fracture surgery	>65 years	40	475	KDIGO classification	Retrospective cohort study
Kang 2020[8]	2011–2016	Korea	Hip fracture surgery	70.1 years	25	550	AKIN classification	Case–control study
Küpeli 2020[23]	January (1–7), 2018	Turkey	Hip fracture surgery	>65 years	28	160	KDIGO classification	Retrospective descriptive study

AKI, Acute Kidney Injury; KDIGO, Kidney Disease Improving Global Outcome; AKIN, Acute Kidney Injury Network

**Table 2.** NOS score of included studies in the meta-analysis

Study	Selection	Comparability	Exposure	Total Score
Craig 2012[16]	2	2	3	7
Ulucay 2012[17]	3	2	3	8
Marty 2016[3]	3	2	3	8
Pedersen 2016[19]	3	2	3	8
Hong 2017[18]	3	2	2	7
Shin 2018[6]	3	2	3	8
Frenkelrutenberg 2019[20]	2	2	3	7
Jang 2019[21]	3	2	3	8
Rantalaiho 2019[22]	2	2	3	7
Kang 2020[8]	2	2	3	7
Küpeli 2020[23]	2	2	2	6

NOS, Newcastle–Ottawa Scale

### Postoperative AKI in patients undergoing hip fracture surgery

**Figure 2.**
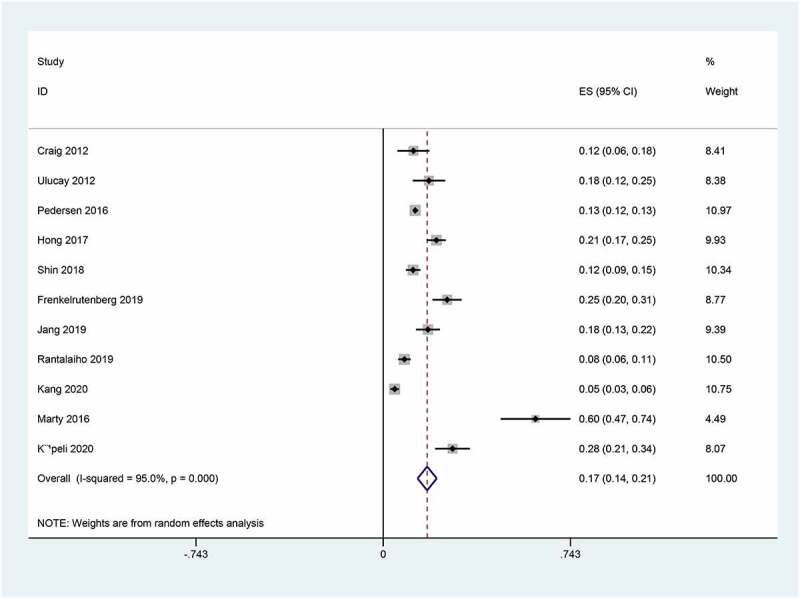
Forest plot for prevalence of AKI in patients undergoing hip fracture surgery using random-effects mode

**Figure 3.**
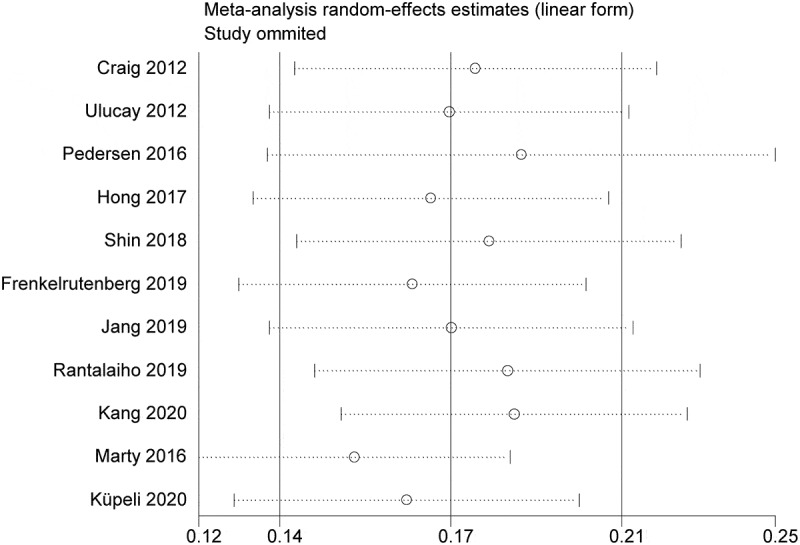
Sensitivity analysis for prevalence of AKI in patients undergoing hip fracture surgery in the meta-analysis

**Figure 4.**
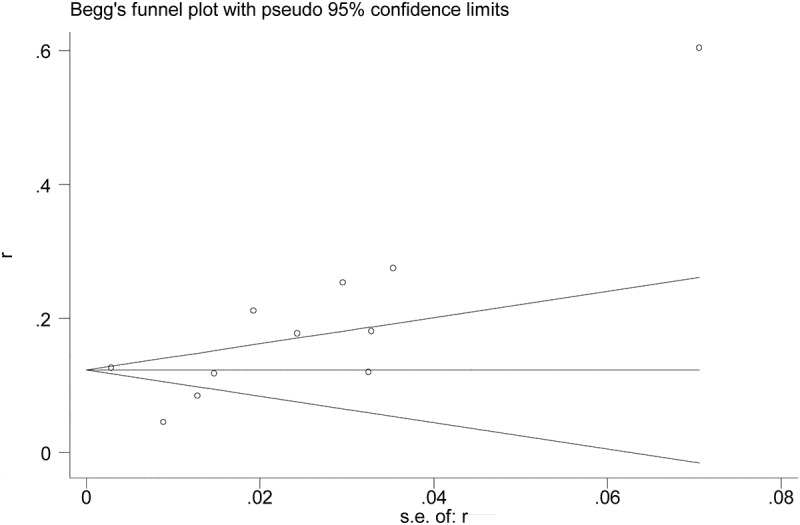
Funnel plot for prevalence of AKI in patients undergoing hip fracture surgery. (Egger’s test, *p* = 0.258 and Begg’s test, *p* = 0.087)

All the included studies reported the prevalence of postoperative AKI in patients undergoing hip fracture surgery. The overall pooled prevalence of AKI after hip fracture surgery was 17% (95%CI, 0.14–0.21). The statistical heterogeneity was significant (I^2^ = 95%) (Figure 2). Subsequently, we performed meta-regressions to explore the potential sources of statistical heterogeneity. The results indicated that publication time (p = 0.368), sample size (p = 0.593), and NOS score (p = 0.558) may not be the potential sources of statistical heterogeneity. Also, we conducted stratified analyses to explore the prevalence of AKI in subgroup patients. In subgroup analyses stratified by region, the prevalence of AKI in Asia (22%) was higher than that in Europe (12%). When stratified by sample size, the prevalence of AKI in sample size >500(40%) was higher than that in sample size ≤500 (15%). In subgroup analysis by study design, the prevalence of AKI in the subgroup of cohort study (14%) was lower than that in other subgroup(43%). Interestingly, the prevalences of AKI in subgroup stratified by AKI definition and NOS score were basically the overall pooled prevalence of AKI. The detailed results of subgroup analyses were showed in Table 3. Furthermore, we undertook sensitivity analysis to explore the influence of individual included studies on the overall pooled estimate. The results of sensitivity analysis revealed that the pooled prevalences of AKI were basically consistent with the overall pooled effect, which indicated that the overall pooled estimate was robust and credible (Figure 3). We further evaluated the potential publication bias using the funnel plot and statistical tests. The funnel plot seemed to be asymmetric, but the statistical results indicated that the publication bias was not statistically significant (Egger’s test, *p* = 0.258 and Begg’s test, *p* = 0.087; Figure 4).

**Table 3.** Subgroup analysis for the prevalence of AKI in patients undergoing hip fracture surgery

Outcomes	Number of trials	Pooled prevalence with 95%CI	I^2^ (%)
Primary analysis	11	0.17(0.14–0.21)	95
Region			
Asia	7	0.22(0.15–0.29)	93.9
Europe	4	0.12(0.07–0.18)	97
Sample size			
>500	2	0.40(0.02–0.79)	96.5
≤500	9	0.15(0.11–0.18)	94
Study design			
Cohort study	9	0.14(0.11–0.18)	94.1
Others	2	0.43(0.11–0.76)	94.3
AKI Definition			
Self-definition	1	0.12(0.06–0.18)	
KDIGO	5	0.17(0.09–0.25)	96.7
AKIN	5	0.20(0.15–0.25)	92.4
NOS score			
>7	5	0.17(0.12–0.21)	92.2
≤7	6	0.20(0.12–0.29)	95.9

AKI, Acute Kidney Injury; CI, Confidence interval; KDIGO, Kidney Disease Improving Global Outcome; AKIN, Acute Kidney Injury Network

### Risk factors for AKI in patients undergoing hip fracture surgery

We also explore the potential risk factors associated with AKI after hip fracture surgery. A total of seven studies reported AKI-associated risk factors on multivariate or adjusted model (Table 4) [3,6,8,17,19,21,22]. In the current meta-analysis, we merely considered risk factors reported in two or more eligible studies for meta-analysis. Of these identified risk factors, two studies reported postoperative serum albumin on multivariable model. The pooled estimate indicated that postoperative serum albumin (two studies; OR 1.80; 95%CI, 1.38–2.36; Table 5) was a significant predictor for AKI in patients undergoing hip fracture surgery. Pooled analysis based on three studies revealed that three studies; OR 1.01; 95%CI, 0.95–1.07; Table 5) did not significantly increase the risk of AKI after hip fracture surgery. Similarly, a pooled analysis based on two studies found that ACE inhibitors (two studies; OR 1.38; 95%CI, 0.92–2.07; Table 5) and estimated glomerular filtration rate (two studies; OR 0.98; 95%CI, 0.91–1.06; Table 5) were not associated with increased risk of AKI in patients undergoing hip fracture surgery.

**Table 4.** Risk factors associated with AKI on multivariate model in patients undergoing hip fracture surgery

Study	Risk factors on multivariate model
Ulucay 2012[17]	Age, years: (OR 1.049, 95%CI 0.984–1.118); Gender (female):(OR 2.643, 95%CI 0.909–7.686); Potassium: (OR 1.688, 95%CI 0.693–4.110); eGFR:(OR 0.945, 95%CI 0.921–0.963)
Marty 2016[3]	Preop RI:(OR 0.03, 95%CI 0.01–75,228);Postop RI:(OR 1.6*10^12^, 95%CI 3779–679*10^18^); GFR Preop:(OR 9.7, 95%CI 0.88–107); Age, years: (OR 0.92, 95%CI 0.84–1.01)
Pedersen 2016[19]	Obese patients for AKI 1 stage(HR 1.4, 95%CI 1.1–1.8), AKI 2 stage(HR 1.9, 95%CI 1.3–3.0), AKI 3 stage(HR 2.8, 95%CI 1.5–4.9)
Shin 2018[6]	Age (years):(OR 1.022, 95%CI 0.983–1.064); Chronic kidney disease:(OR 3.879, 95%CI 1.885–7.981);ACE inhibitors(OR 1.751, 95%CI 0.928–3.302);NSAIDs(OR 0.718, 95%CI 0.339–1.291); Koval score(OR 1.067, 95%CI 0.916–1.244);Postoperative serum albumin(OR 1.972, 95%CI 1.029–3.779); Postoperative drained blood volume(OR 1.003, 95%CI 0.999–1.007)
Jang 2019[21]	Type of operation(OR 0.33, 95%CI 0.09–0.94); Diabetes mellitus:(OR 2.36, 95%CI 0.80–7.01);Previous renal disease(OR 2.57, 95%CI 0.60–3.24);ACE inhibitor(OR 1.43, 95%CI 0.50–1.17); Hemoglobin(OR 1.43, 95%CI 0.50–1.17);BUN(OR 1.03, 95%CI 0.99–1.08);eGFR(OR 1.02, 95%CI 0.99–1.04); Intraoperative hypotension(OR 5.14, 95%CI 1.54–20.35)
Rantalaiho 2019[22]	Dementia(RR 2.37, 95%CI 1.00–4.98); Preoperative sCr:(RR 1.01, 95%CI 1.01–1.02)
Kang 2020[8]	Hospitalization(OR 1.24, 95%CI 0.96–1.57);EBL(OR 1.54, 95%CI 1.32–2.44);Postoperative serum albumin(OR 1.77, 95%CI 1.52–2.74)

AKI, Acute Kidney Injury; OR, odds ratio; CI, Confidence interval; eGFR, estimated glomerular filtration rate; GFR: glomerular filtration rate; preop RI: preoperative doppler renal resistive index; postop RI: postoperative doppler renal resistive index; HR, hazard ratio; ACE inhibitors = angiotensin-converting enzyme inhibitors; NSAIDS = Non-steroidal anti-inflammatory drugs; BUN, blood urea nitrogen; EBL: estimated blood loss

**Table 5.** Meta-analysis of risk factors for AKI in Patients undergoing hip fracture surgery

Outcomes	Number of trials	OR (95% CI)	I^2^(%)
Age	3	1.01(0.95–1.07)	63.7
ACE inhibitors	2	1.38(0.92–2.07)	0
Postoperative serum albumin	2	1.80(1.38–2.36)	0
eGFR	2	0.98(0.91–1.06)	95.1

AKI, Acute Kidney Injury; OR, odds ratio; CI, Confidence interval; ACE inhibitors, angiotensin-converting enzyme inhibitors; eGFR, estimated glomerular filtration rate

## Discussion

The current meta-analysis revealed that AKI was a relatively frequent complication in patients undergoing hip fracture surgery with pooled prevalence ranging from 14% to 21%. Additionally, postoperative serum albumin was identified to be a significant risk factor for AKI following hip fracture surgery.

The current meta-analysis based on 11 observational studies indicated that the overall pooled prevalence of AKI following hip fracture surgery was 17% with substantial heterogeneity. Considering that the significant heterogeneity may impair the credibility of the pooled estimate, meta-regression was performed to explore the potential sources of statistical heterogeneity. Furthermore, we found that publication time, sample size, and NOS score may not be responsible for significant statistical heterogeneity. Subsequently, we conducted subgroup analysis and sensitivity analysis to explore the prevalence of AKI in sub-population. Interestingly, the results of subgroup analysis and sensitivity analysis were basically consistent with the overall pooled effect, which suggested that the overall pooled estimate was robust and reliable. A previous meta-analysis showed that the overall estimated prevalence rates of AKI in patients undergoing total hip arthroplasties are 6.3%[5]. Obviously, the prevalence of AKI following total hip arthroplasties was lower than that in patients undergoing hip fracture surgery. Regardless of the fact that the exact causes for these differences were largely unclear, but surgical workers should attach more importance to the potential AKI in patients undergoing hip fracture surgery. In the study, we also investigated the risk factors for AKI following hip fracture surgery. Pooled analysis showed that postoperative serum albumin was a significant indicator for AKI in patients undergoing hip fracture surgery. Consistent with our results, some previous studies also found that serum albumin level was a potential risk factor for AKI. Thongprayoon et al. revealed that there existed a U-shape correlation between serum albumin levels and AKI in hospitalized patients[24]. Dos Santos and coworkers found that low serum albumin concentration was associated with increased risk of AKI in critically ill patients[25]. Mechanically, a recent study found that 5-Lypoxygenase products induced by albumin overload may be responsible for renal tubulointerstitial injury[26]. Other risk factors including age, ACE inhibitors, and eGFR were possible predictors for AKI, although the pooled results were not statistically significant. Collectively, clinicians should pay attention to these identified risk factors, which may contribute to preventing or decreasing the risk of AKI after hip fracture surgery.

The current study is the first meta-analysis which systematically investigated the prevalence and risk factors after hip fracture surgery. An important strength of our study lies in its accordance MOOSE checklist and PRISMA guidelines. Two reviewers independently conducted literature search, data extraction, quality assessment, and statistical analysis, which facilitate the transparency and replicability of the meta-analysis. There also existed several limitations in the current study. First, our meta-analysis showed substantial statistical heterogeneity, which may potentially impair the reliability of the pooled estimate. Subsequently, we performed meta-regression to explore the sources of statistical heterogeneity and none of the significant factors was identified to be responsible for significant heterogeneity. A possible interpretation is that multiple clinical and methodological differences across included studies, but not individual factors, contribute to the significant statistical heterogeneity. Irrespective of the statistical heterogeneity, the results of subgroup analysis and sensitivity analysis were basically consistent with the overall pooled effect, which showed the robustness and reliability of the pooled estimate. Second, we evaluated the potential publication bias using the funnel plot and statistical tests. The statistical results showed that the publication bias was not statistically significant, but the funnel plot seemed to be asymmetric. Considering the inconsistency, the potential publication bias still cannot be excluded, although we performed a systematic literature search in the meta-analysis. Third, some risk factors reported in included studies were not pooled for meta-analyses owing to limited studies, which may bias the authentic effects for AKI. The pooled analysis based on two studies found that ACE inhibitors may not be a significant risk factor for AKI following hip fracture surgery. Actually, many studies found that ACE inhibitors were a significant predictor for AKI [27–29]. Also, many risk factors including chronic kidney disease, intraoperative hypotension, and dementia were reported to be significant predictors for AKI, but we did not include them for further pooled analyses owing to the fact that they were reported in the limited studies. Therefore, the limited studies may bias the authentic estimates in the current meta-analysis. Accordingly, the results in our meta-analysis may be relatively conservative and should be interpreted with caution.

## Conclusions

The current meta-analysis revealed that the pooled AKI in patients undergoing hip fracture surgery was approximately 17%. Postoperative serum albumin was identified as a potentially significant risk factor for AKI. Further high-quality studies should be warranted to systematically clarify the prevalence and risk factors of AKI following hip fracture surgery.

## Highlight

1. The pooled prevalence of AKI following hip fracture surgery was approximately 17%.

2. Postoperative serum albumin was a significant risk factor for AKI following hip fracture surgery.

3. Age and ACE inhibitors may be potential risk factors of AKI following hip fracture surgery.

## Abbreviations

AKI: Acute kidney injury; CI: Confidence interval; OR: odds ratio
